# Optimization of Ultrasonic Extraction to Obtain Erinacine A and Polyphenols with Antioxidant Activity from the Fungal Biomass of *Hericium erinaceus*

**DOI:** 10.3390/foods9121889

**Published:** 2020-12-18

**Authors:** Mihai-Vlad Valu, Liliana Cristina Soare, Nicoleta Anca Sutan, Catalin Ducu, Sorin Moga, Lucian Hritcu, Razvan Stefan Boiangiu, Simone Carradori

**Affiliations:** 1 Department of Natural Sciences, Faculty of Science, University of Pitesti, Targu din Vale Street, 110040 Pitesti, Romania; mihai.valu@upit.ro (M.-V.V.); cristina.soare@upit.ro (L.C.S.); anca.sutan@upit.ro (N.A.S.); 2 Regional Research and Development Center for Innovative Materials, University of Pitesti, Products, and Processes from Automotive Industry, 11 Doaga Street, Arges, 110440 Pitesti, Romania; catalin.ducu@upit.ro (C.D.); sorin.moga@upit.ro (S.M.); 3 Department of Biology, Alexandru Ioan Cuza University of Iasi, Bd. Carol I, No. 11, 700506 Iasi, Romania; hritcu@uaic.ro (L.H.); razvan.boiangiu@student.uaic.ro (R.S.B.); 4 Department of Pharmacy, “G. d’Annunzio” University of Chieti-Pescara, Via dei Vestini 31, 66100 Chieti, Italy

**Keywords:** *Hericium erinaceus*, ultrasonic extraction, erinacine A, HPLC analysis, antioxidant activity

## Abstract

*Hericium erinaceus* is a medicinal fungal species that produces the active biological metabolite erinacine A with strong antioxidant activity. The classical extraction techniques used to date to obtain metabolites from this fungal species require high consumption of resources and energy and, in the end, prove to be expensive and inefficient, especially on a biomedical scale. The aim of this research is based on the development of an ultrasonic extraction (UE) method for the identification and extraction of biological compounds with high antioxidant activity from the mycelia of *H. erinaceus* biomass developed through a solid cultivation process. The extraction process was optimized by varying parameters to determine the best extraction yield of metabolites involved in such antioxidant activity, using the response surface methodology (RSM). The physicochemical analyses were oriented towards the investigation of polyphenols, flavonoids, and the diterpenoid erinacine A. It is highlighted that there is a very good mutual connection between the concentration of polyphenols and flavonoids in the extracts studied and the diterpenoid erinacine A. Also, this study describes an efficient and qualitative extraction method for extracting natural antioxidants from the *H. erinaceus* mushroom, since toxic solvents were not used in the developed extraction procedure. This biomass can be used both as a food source and as a possible phytotherapeutic tool in the prevention or treatment of various neurodegenerative disorders that require drugs with strong antioxidant activity.

## 1. Introduction

**Figure 1:**
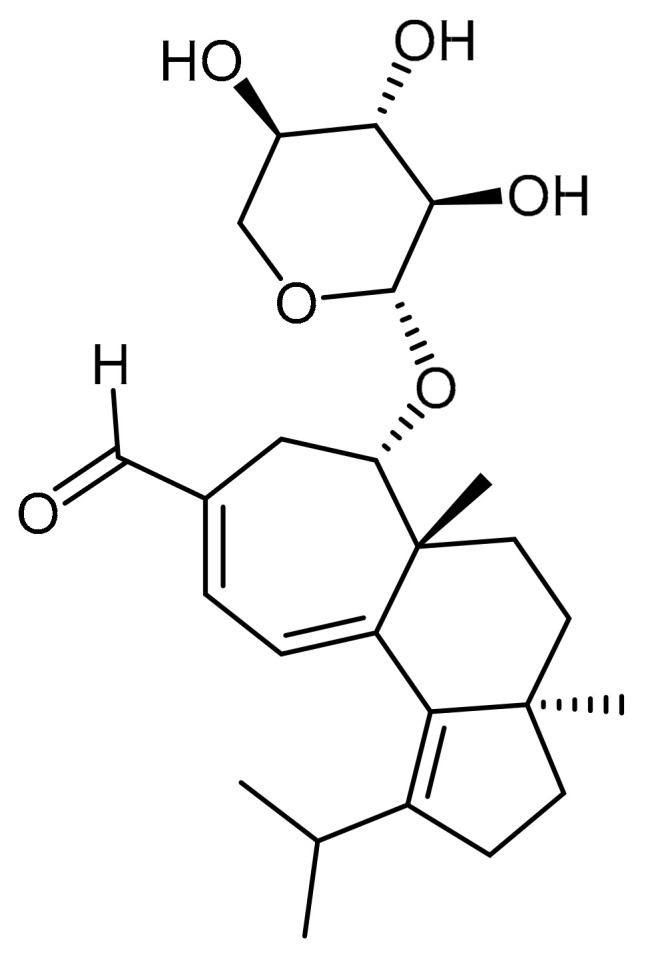
Chemical structure of erinacine A. Chemical Formula: C_25_H_36_O_6_ (432.5 g/mol.).

Certain edible mushrooms can be classified as medicinal, having many biologically active compounds for human health, due to their consistency [1]. Before the advancement of modern medicine, people relied on natural remedies with antioxidant activity for the prevention and/or treatment of various diseases, including Parkinson’s disease, Alzheimer’s disease (AD), cancer, etc. [2]. The therapeutic potential of secondary metabolites in mushroom species has received special attention recently, especially regarding neurodegenerative diseases such as Alzheimer’s disease [3,4]. For example, in the case of AD, no safe means of prevention is known, and it is a progressive neurodegenerative disorder with etiopathogenesis involving the participation of numerous risk factors [5]. It is important to point out that extraction is considered the first step in advancing the development of the main traditional mushroom-based remedies. Considerable efforts have been made by researchers to find the most effective extraction methods having better efficiency and providing extracts endowed with high bioactivity [6,7]. It should be noted that attempts are stilll being made to develop an extraction method that is suitable for mushrooms, applying different parameters for high efficiency [8]. Many published studies have reported that ultrasonic extraction can improve the extraction rate of active biological heat-sensitive ingredients under low-temperature processing conditions, making it more effective than conventional extraction techniques [9]. The mechanical effect of ultrasonication causes the penetration of solvents into tissue cells, improved mass transfer, and tearing of the cell wall, which is favorable to the release of tissue cell content and can reduce the processing time and required amount of solvents compared to traditional extraction methods [10]. *Hericium* is a genus of edible mushrooms of the Hericiaceae family. The genus *Hericium* produces phytochemical substances, erinacines and hericenones, with antioxidant activity, but also various polysaccharides [11]. Erinacine A (Figure 1) was isolated from the fungal species *Hericium erinaceus*; its chemical structure is deposited in PubChem with the indication 9,867,477.

Mainly, the antioxidant activity of the *H. erinaceus* species is due to the presence of polysaccharides (β-glucans) and diterpenoids (hericenons, erinacines), but several authors have stated that there is a close link between the antioxidant activity of *H. erinaceus* and the presence of flavonoids and polyphenols [12,13]. It has been noted that the biosynthesis of erinacine A in submerged culture can lower production costs because the chemical synthesis of diterpenoids is a long, multistage process and provides poor yield [13,14]. Most scientific studies on the antioxidant properties of *H. erinaceus* have described classical extraction techniques, although in the literature there are different modern methods of extraction. Therefore, an additional task of this study was to investigate the antioxidant properties of *H. erinaceus* extracts obtained by ultrasonic extraction. At the same time, given that study results have been contradictory concerning the presence of flavonoids and polyphenols with antioxidant activity in mushroom species [15,16], this issue has aroused our interest to investigate various physicochemical methods for the possible presence of these biocompounds. Besides this, the number of components with antioxidant effects was determined. It has been shown that the antioxidant effects of several mushrooms are also related to their total phenolic and flavonoid contents [17]. Further, it is expected that the antioxidant activity is due to the high yield of extracted erinacine A as identified by HPLC. The extraction parameters (extraction time, ethanol concentration, and solvent/material ratio) were also investigated to maximize the extraction yield in terms of the number of metabolites and to establish optimal extraction conditions. The response surface method (RSM) is an innovative statistical method used to optimize processing parameters. Therefore, RSM is used to speed up and optimize operating processes to reduce the required time, energy, and raw materials. The response surface methodology was applied to optimize the ultrasonic extraction parameters to obtain high yield and content of antioxidant compounds of HE. The main advantage of RSM is that it can reduce the required number of experiments and highlight the relationship between response and variables. This is the first report on the determination of erinacine A, as well as on the optimization of the ultrasonic extraction conditions of metabolites with antioxidant activity. In this work, we aimed to enhance the phenolic, flavonoid, and erinacine A contents and antioxidant activity of *H. erinaceus* biomass under ultrasonic extraction.

## 2. Materials and Methods

### 2.1. Chemicals and Reagents

Erinacine A, not commercially available, was obtained from the fungal biomass of *H. erinaceus* as previously reported [18]. Casein peptone, glucose, sodium chloride (NaCl), monopotassium phosphate (KH_2_PO_4_), hydrochloric acid (HCl), and nitric acid (HNO_3_) were obtained from ThermoFisher Scientific (Darmstadt, Germany). Folin–Ciocalteu reagent, 2,2-diphenyl-1-picrylhydrazyl (DPPH), quercetin, gallic acid, L-ascorbic acid, galantamine, acetylthiocholine iodide, butyrylthiocholine chloride, trichloroacetic acid (TCA), and iron(III) chloride (FeCl_3_) were all purchased from Sigma-Aldrich (Steinheim, Germany), and commercial ethyl alcohol (96%) was purchased from Prodvinalco S.A. Romania. The other substances were of analytical quality.

### 2.2. Solid-State Cultivation of H. erinaceus Biomass

In this research, the *H. erinaceus* biomass was developed on a solid culture medium under controlled conditions to obtain the diterpenoid erinacine A, and the antioxidant activity was investigated. The biomass was developed in the laboratory University of Agronomic Sciences and Veterinary Medicine of Bucharest (USAMV, Bucharest) for 21 days following an adapted protocol [19]. In a 9.0 L glass vessel, the culture medium was prepared from casein peptone 12.16 g/L, glucose 66.88 g/L, NaCl 1.46 g/L, and KH_2_PO_4_ 1.0 g/L with a pH of 4.5 [19]. Moreover, to enrich the yield of the compounds of interest, namely, erinacine A, flavonoids, and polyphenols, contents of 0.5% yeast extract, 4% glucose, 0.5% soy powder, and 1% oats were added to the culture medium.

### 2.3. Ultrasonic Extraction Procedure

The technology for obtaining and concentrating the bioactive products of the *H. erinaceus* biomass was developed based on the principles of the disintegration of cell structures using ultrasound waves. The device used for extraction was a Hielscher ultrasonic processor (Hielscher UIP1000hdT Berlin, Germany), with a sonotrode of 40 mm diameter, 1000 Watts, 20 kHz, and adjustable amplitude (amplitude ratio 1:0.7). Before the extraction experiments, the ultrasonic processor was calibrated to find the power consumption of the equipment. During the sonication process, this value was automatically deducted from the gross energy consumption, thus allowing the net power delivered to the extraction medium to be found. During the experiments, the samples were placed in an ice bag with continuous magnetic stirring to maintain a low sample temperature. After completion of the extraction, the samples were vacuum filtered and then centrifuged (2500× *g* for 5 min). A rotary evaporator (Heidolph Hei-VAP Core, Schwabach, Germany) was used for water and alcohol elimination from the supernatants. The remaining water and alcohol residues from the samples were subjected to the lyophilization process.

### 2.4. Lyophilization of Samples

The last stage consisted of lyophilization of the samples (Christ Alpha 1–2 LDplus, Osterode, Germany) to obtain a powder (permanently prepared, shredded as particles) of the fungal material. The aim of the lyophilization was to obtain high-quality extracts to maintain extraction efficiency, with a higher content in active principles due to freeze-drying [20]. The working procedure was optimized and consisted of subjecting the liquid samples in the Petri dishes (7 mm) to freezing at the vaporization temperature of −70 °C for 2 h and freeze-off at −55 °C under vacuum for 48 h. Vacuum in the enclosure was achieved at a constant pressure of 400 μBar. Finally, the material was restored to room temperature, resulting in a powder extract. An important aspect of freeze-making is that it limits oxidative changes in metabolites since the oxygen concentration is very low under vacuum.

### 2.5. Optimization of the Extraction Procedure Using the RSM Model

Response surface methodology (RSM) was applied to investigate the impact of three independent variables on the total phenolics and yield of *H. erinaceus* extract. The factors that influence the extraction quality are solvent concentration (%, X1), extraction time (min, X2), and the solvent–material ratio (mL/g, X3). The temperature was not taken into account in this experimental model because the extraction time started once the desired temperature was reached. The experiments were performed using the Box–Behnken Design (BBD). Three variables were taken into consideration to optimize the best combination of extraction parameters for extract yield and total phenolic content. The complete design was performed in random order, involving 17 experiments, including 5 replicates at the central point (Table 1). The analyses were determined by the following formula:

$$Y=\beta_{0}+\;\sum b_{i}X_{i}+\sum b_{ii}X_{i}^{2}+\sum b_{ij}X_{i}X_{j}.$$

The quality of the fitted model was determined by the coefficient of determination (R). A central composite design (CCD) was carried out to study the effects of the three independent variables.

### 2.6. Proximate Composition

Samples were evaluated using AOAC (The Association of Official Analytical Chemists) procedures with minor modifications [21]. The total carbohydrate content was measured by subtracting the contents of moisture, ash, fat, and protein from 100 and reported as a percentage of dry mass. The dietary fiber content was determined by enzymatic and gravimetric methods. The crude protein content was evaluated by the Macro-Kjeldahl method.

### 2.7. Macro- and Microelements

Determination of the mineral content (ash) and analysis of the mineral elements were made on dry samples via the AOAC method no. 930.05. The incineration residue was extracted with 0.5 mL/mL HCl and 0.5 mL/mL HNO_3_, then distilled water was added and Fe, Cu, Mn, and Zn were weighed directly [22,23]. Standard solutions were used to compare absorption responses with standard analytical solutions of purity >99.9%.

### 2.8. Reducing Power

The reducing power of the ultrasonic extract was determined according to an established method [24]. Increased absorbance of the reaction mixture indicated increased reducing power. Evaluation of the reduction potency was performed by mixing each extract in different solutions. The mixture was incubated at 50 °C for 20 min. Trichloroacetic acid (TCA, 10%, 2.5 mL) was added to a portion of this mixture (5 mL) and centrifuged at 3000× *g* for 10 min. The supernatant was separated and mixed with distilled water (2.5 mL) containing 1% ferric chloride (0.5 mL). The absorbance was measured at 700 nm (Ocean Optics HR2000+ Spectrometer, Ostfildern, Germany).

### 2.9. Determination of the Total Phenol and Flavonoid Contents of H. erinaceus Extract

The flavonoid content was measured according to the method described by Delcour and Varebeke [25], and quercetin was used for the analytical curve. The results are expressed as milligrams of quercetin equivalent per gram of dry matter of mushroom extract (mg QE/g DM). After an incubation period of 10 min, the absorbance was read at 640 nm. The total phenolic contents of the extracts were calculated using Folin–Ciocalteu reagent, following the method of Velioglu [26], where gallic acid (mg GAE/g DM) was used as a standard antioxidant. The analysis was performed using an Ocean Optics HR2000+ Spectrometer (Ostfildern, Germany).

### 2.10. DPPH-Radical Scavenging (Antioxidant) Activity

The free radical scavenging rate was determined by measuring the 2,2-diphenyl-1-picrylhydrazyl (DPPH) with L-ascorbic acid as a reference standard. For the DPPH assay, we used a modified procedure described in a previous study [27]. The lyophilized powder extract from *Hericium erinaceus* was dissolved in methanol (2 mL extracts and 2 mL Tris-HCl buffer) and mixed with 260 μL of a 0.2 mM DPPH radical solution (Sigma-Aldrich, St. Louis, MO, USA). The percentage inhibition was calculated according to the formula [(A_0_ − A_1_)/A_0_] × 100, where A_0_ is the absorbance of the control and A_1_ is the absorbance of the sample.

### 2.11. Determination of Acetylcholinesterase (AChE) and Butyrylcholinesterase (BChE) Inhibitory Activities

The AChE and BChE inhibitory activities were measured by slightly modifying the spectrophotometric method [28]. Electric eel AChE (Type-VI-S, EC 3.1.1.7, Sigma Aldrich/MERK, Milan, Italy) and horse serum BChE (Sigma Aldrich/MERK, Milan, Italy) were used, while acetylthiocholine iodide and butyrylthiocholine chloride (Sigma Aldrich/MERK, Milan, Italy) were chosen as the substrates of the reaction. Galantamine (Sigma Aldrich/MERK, Milan, Italy), was used as the reference drug [29].

### 2.12. HPLC/DAD-UV Analysis

HPLC analysis of erinacine A was conducted according to a previous study [18]. The retention time of erinacine A was approximately ~17.8 min at a flow rate of 1.0 mL/min at 380 nm. Erinacine A was used as a standard as previously reported [30]. A stock solution (1 mg/mL) of erinacine A was prepared in 70% ethanol. Standard solutions of erinacine A within the final concentration range of 1–25 µg/mL were prepared (see Figure S1 for the calibration curve).

### 2.13. Statistical Analysis

The experimental results of the RSM were analyzed using Matlab2018b software. *p*-Values less than 0.05 were considered to indicate statistical significance. The data were analyzed using GraphPad Prism 7 software, and the HPLC data were analyzed using OriginPro software.

## 3. Results

### 3.1. Effect of Different Ultrasonic Parameters on the Yield of H. erinaceus Extracts

**Figure 2:**
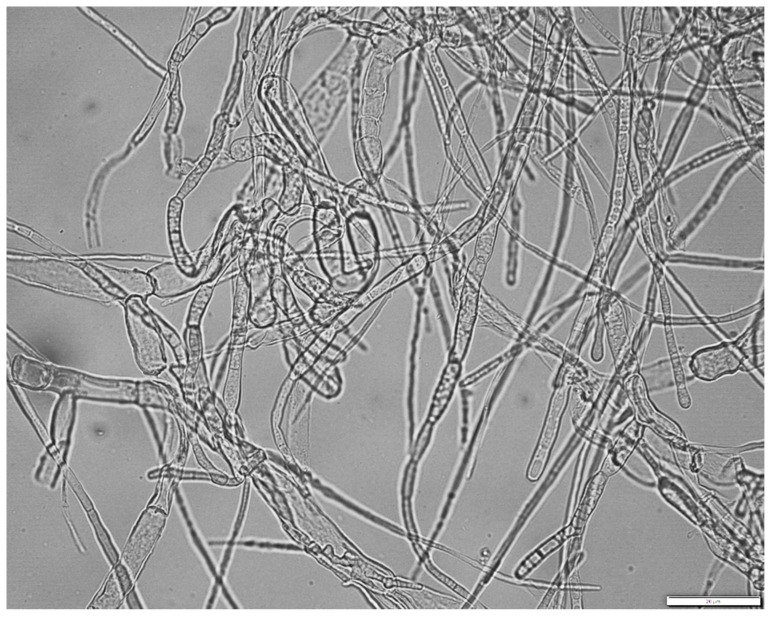
Mycelial filamentous hyphae developed inside the biomass of *Hericium erinaceus* (Olympus BX43, Hamburg, Germany). Scale bar: 20 µm.

A microscopic analysis (Olympus BX 43, Hamburg, Germany) was performed to confirm the development of *H. erinaceus* biomass in the culture vessel and observe the presence of mycelial hyphae (Figure 2), and images were taken with the digital camera of the Olympus Bx 43 instrument. The analysis was performed with white LED lamp housing (U-LHLEDC), with a 40x objective lens and a 10-fold ocular, using an Olympus DP72 camera sensor (Olympus Corporation, Hamburg, Germany) (11.8 megapixel cooled digital color camera, capturing each color of RGB at 12 bits, at ISO 1600 setting, image color-calibrated (XC30 software)). The parameters tracked during ultrasonic extraction by sonochemistry were approximately (extraction time being different) similar for each sample examined and were constantly monitored from the operating system of the device using 27 W per hour, with a net extraction power of 170 W, and maintaining a constant temperature in the ultrasonic bath.

It should be specified that temperature and time were controlled in order not to degrade biological compounds in the *H. erinaceus* biomass, and the former was maintained constant by circulating external cold water and using a T-type thermocouple. However, the temperature of the ultrasonic bath during the entire sonication period was kept as constant as possible by the addition of ice sheets. In general, ultrasonication increases the rate of a chemical reaction; the reaction conditions are easier to meet, and the energy consumed can be reduced [10]. To achieve a more exhaustive procedure, it was important to investigate the process variables. Preliminary experiments enabled the range of ethanol concentrations (40–80%), extraction time (20–45 min), and solvent-to-material ratio (10–30 mL/g) to be fixed. It can be seen in Table 1 and Table 2 that the extraction yield was affected most significantly by ethanol (X_1_) (*p* < 0.05) and the solvent-to-material ratio (X_3_), followed by extraction time (X_2_) (*p* < 0.05). The quadratic parameter X_1_^2^ was significant at the level of *p* < 0.05, whereas the two quadratic parameters (X_2_^2^, X_3_^2^) and interaction quadratic parameters (X_1_X_2_, X_2_X_3_, X_1_X_3_) were non-significant (*p* > 0.05) with respect to extraction yield.

### 3.2. Response Surface Method (RSM)

**Figure 3:**
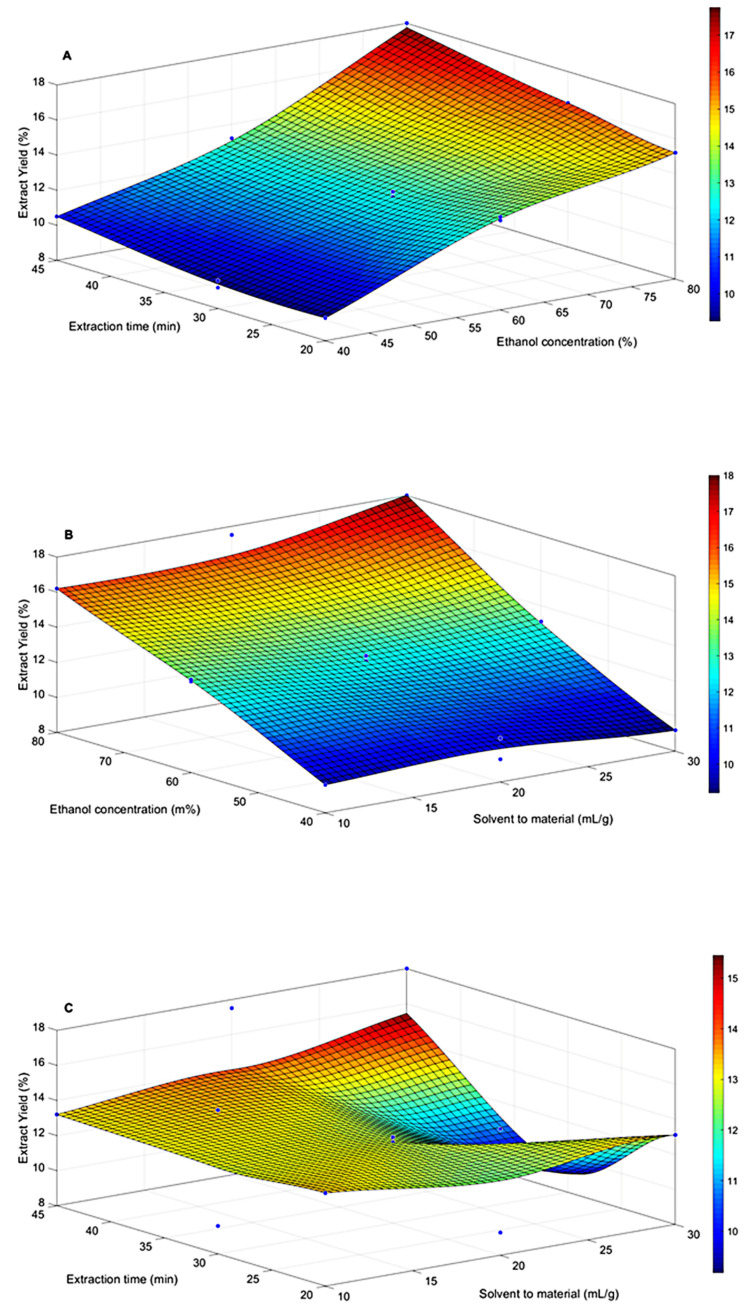
Response surface analysis for the extract yield (%) via ultrasonic extraction (UE) for (**A**) extraction time and ethanol concentration; (**B**) ethanol concentration and solvent-to-material ratio; (**C**) extraction time and solvent-to-material ratio. The data were analyzed using Matlab software.

Figure 3 displays the interactions among the ethanol concentration and each of the two other factors, namely, extraction time and solvent-to-material ratio; the effect of their mutual interaction on the extraction yield of *H. erinaceus* can thus be extrapolated.

In Figure 3A is shown the amplitude of the interaction between ethanol concentration (X_1_) and extraction time (X_2_) regarding the extraction yield. Increasing percentages of ethanol from 60 to 80% with extraction time from 30 to 45 min enhanced the extraction yield. It can be seen in Figure 3B that by varying the ethanol concentration from 60 to 80% and increasing the solvent-to-material ratio from 10 to 30 mL/g, the extraction yield of target compounds was improved. Figure 3C presents the correlation between extraction time and the solvent-to-material ratio. It was found that the maximum yield (17.50%) was achieved when the extraction time was 45 min and the solvent-to-material ratio was 20 mL/g. This result was in accordance with the findings of previous studies [31,32].

### 3.3. Effect of Extraction Parameters on TPC, TFC, and DPPH

The total phenolic content in the extract is expressed in milligrams of gallic acid equivalent and the total flavonoid content in milligrams of quercetin equivalent. In this study, however, the total phenolics yield decreased when the ethanol concentration was more than 80% (Table 1). The total phenolics yield increased with prolonged extraction time from 20 to 45 min. The total phenolics yield increased with an increase in ethanol concentration from 60% to 80%. It should be noted that, although in a very small amount, flavonoids are also present in the extract of *H. erinaceus*. The ethanol extracts exhibited high content of phenolics and high radical scavenging activity (DPPH scavenged, as a percentage). The results strongly suggest that phenolics and flavonoids are important components of the *H. erinaceus* extracts, and this could explain their high radical scavenging activity. The total flavonoid (TFC) and phenolic (TPC) contents of *H. erinaceus* were quantified using the Folin–Ciocalteu reagent (Table 1). It was found that the total phenolic content of *H. erinaceus* reached 23.26 mg GAE/g DM and the total flavonoid content reached 3.26 mg QE/g DM with 80% ethanol concentration, extraction time of 45 min, and solvent–material ratio 30 mL/g, these data being consistent with those from other scientific studies [12,33]. In the DPPH assay, the maximum antioxidant activity of *H. erinaceus* corresponded to an IC_50_ of 92.4 µg/mL. The results of the present study showed that a dose-dependent increase in free radical extinction is due to the increase in alcohol concentration (80%) with the lowest IC_50_ value in this assay, as shown in Table 1.

### 3.4. Antioxidant Capacities of H. erinaceus Ultrasonic Extracts Concerning Total Phenolics and Other Compounds

The phenols in mushroom extracts can be linked to their antioxidant capabilities. Our research highlights a correlation between total phenolics and DPPH free radical scavenging activity in mushroom extracts. This is consistent with the research of several authors who reported that the total phenolic content correlated with the free radical scavenging activity of other fungi [34,35]. As determined by biochemical methods, the antioxidant activity expressed by the percentage inhibition of the lipid peroxidation reaction was the highest in *H. erinaceus* 80% ethanolic extract. The results confirmed a correlation between these remarkable antioxidant activities and a high content of compounds with free radical scavenging properties.

### 3.5. Proximate Composition

The results of the quantitative analyses to determine the percentages of protein, fat, fiber, carbohydrate, and ash in the bioreactor-developed *H. erinaceus* mycelia are reported in Table 3.

### 3.6. Macro- and Microelements

The mineral composition usually reflects the growth conditions of the mycelia. In other scientific articles, it has been found that medicinal mushrooms contain many mineral elements [16,36]. Our results are highlighted in Table 4. Other researchers mentioned that K is the main macroelement in mushrooms [37], and this was also found in our research.

### 3.7. Reducing Power

The formation was monitored spectrophotometrically at 700 nm. In this study, the scavenging effect of *H. erinaceus* was limited and was found to be inferior, even at higher extract concentration, to that of three standard antioxidants (Table 5).

The investigated mushroom dry extract possessed reductive capabilities, although inferior to ascorbic acid, quercetin, and butylated hydroxyanisole, used as standard antioxidants at the same concentration (0.50 mg/mL). The reducing powers of the extracts produced by ultrasonication with ethanol solvent gave high levels of absorbance. Thus, discrete levels of antioxidant compounds were highlighted.

### 3.8. AChE and BChE Inhibitory Activity

Because of the previous experiments that revealed high antioxidant activity of *Hericium erinaceus* extract obtained using an ethyl alcohol concentration of 80%, we wanted to further check the possible effect on acetylcholinesterase and butyrylcholinesterase to achieve multitarget action for the treatment of AD. Table 6 reports the levels of acetylcholinesterase and butyrylcholinesterase after treatment with the *H. erinaceus* extracts compared with those for galantamine. As shown in Table 6, all extracts showed mild butyrylcholinesterase and acetylcholinesterase inhibitory activities. The 80% alcohol extracts of *H. erinaceus* demonstrated higher inhibitory activity, giving inhibition percentages of up to 53% against AChE and 49% against BChE at 1 mg/mL.

### 3.9. HPLC/DAD-UV Analysis

**Figure 4:**
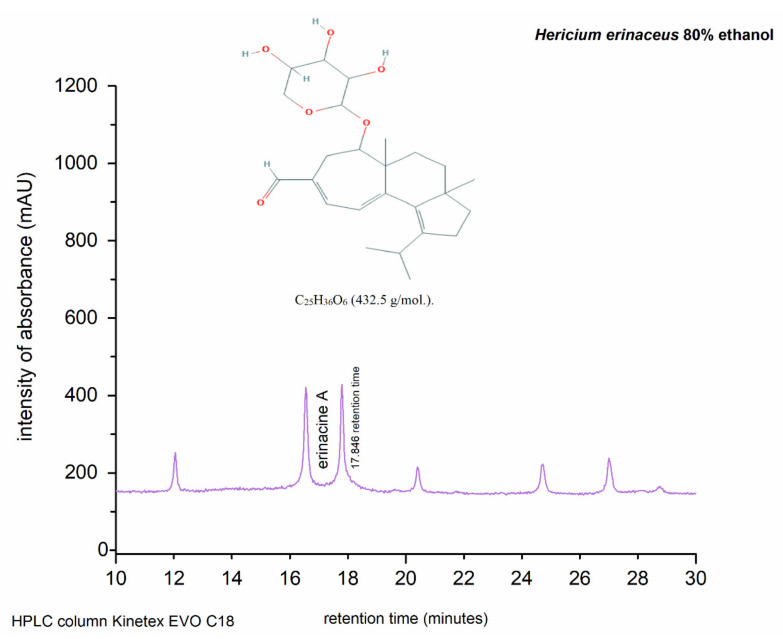
The HPLC sample chromatogram of erinacine A (obtained from *H. erinaceus* mycelium powder extract). The retention time of the diterpenoid erinacine A peak was within the range of 17.846–17.852 min.

Erinacine A was obtained in the glucose-containing medium, as described by [38]. Our work describes the validation of an HPLC/DAD-UV method for the determination of the biocompound erinacine A from the extract of *Hericium erinaceus* (solid-state cultivation) obtained by ultrasonic extraction. The representative HPLC chromatogram of the powder extract of *H. erinaceus* mycelia is shown in Figure 4. The retention time at 17.846 represents erinacine A, one of the most characteristic compounds found in general in *Hericium* species [39].

## 4. Discussion

Past research has shown that the biological activity of fungi can be attributed to polysaccharides, high-molecular-weight glucans [40], and high contents of polyphenols and flavonoids, which are responsible for cytotoxic and antioxidant activities [41,42]. In order to improve the production and the recovery of such bioactive compounds, as well as the content of erinacine A, we modified the development conditions of *H. erinaceus* mycelia in a bioreactor. Then, we used ultrasonication to improve the efficiency of extraction. It is important to also note the fact that not all extracts could furnish a dry product in powder form [20]. Some extracts, particularly non-polar extracts, might lead to an oily product, so consideration should be given to optimizing the extraction method. In our experiments, we can state that our procedure led to a powdered fungal material that maintained and preserved the analyzed biocompounds, as well as the valuable contents of micro- and macroelements. It is known that the lyophilization technique maintains the highest amount of antioxidant compounds compared to other methods of drying extracts. These results were confirmed by previously published studies that showed that the lyophilization process is the most effective way to maintain a high amount of flavonoids in fungi [35]. The differences from other authors [15,16] were likely due to the different conditions of development in the bioreactor of the biological material and the extraction process with ultrasonication.

Given the follow-up, it can be said that a large amount of *H. erinaceus* powder was obtained from a small amount of fungal material. Also, the improvement of the extraction of polyphenols and flavonoids by ultrasonication can mainly be attributed to the effect of acoustic cavitations produced in the solvent by the passage of ultrasonic waves. Therefore, the structure of the cell wall is interrupted, and diffusion through the membranes is accelerated. The most efficient extraction period for achieving the maximum yield was approximately 45 min. However, other parameters could be considered for optimizing the extraction yields of diterpenoids and polyphenols, such as the ultrasonic amplitude of the precipitation time. Based on our results, we can say that the *H. erinaceus* mycelia developed in the culture vessel after our optimized process could improve the extraction of polyphenols, flavonoids, and diterpenoids. This can also be attributed to ultrasonic extraction.

The ANOVA outcome on our extraction conditions, shown in Table 2, revealed that the first-order terms of independent variables (X_1_, X_2_, and X_3_), quadratic terms (X_1_^2^, X_3_^2^, and X_2_^2^), and interaction terms (X_1_X_2_, X_1_X_3_, and X_2_X_3_) mostly affected the content of total phenolics recovered for *H. erinaceus* (*p* < 0.05). This aspect can be explained by the prolongation of the extraction time, which accelerates the chemical decomposition of the bioactive compounds in the extraction process, resulting in lower extraction efficiency. Multiple regression analysis was performed on the data of response variables, such as the DPPH, TPC, and TFC content, as affected by the extraction conditions. Other authors [43,44,45,46,47] also documented positive correlations between total phenolics and DPPH radical scavenging activity, but also the antioxidant potential of mushrooms, due to the presence of the diterpenoid erinacine A. The results indicate that healthy ingredients can be maximally extracted using the optimal solvents and parameters for mushroom extractions, thus modulating the difference in pharmacological effects. Indeed, our results indicated that there was a correlation between DPPH inhibitory activity and total phenolics due to the application of ultrasonic extraction. Thus, from this experiment we concluded that *H. erinaceus* can control oxidative damage and can act as an antioxidant.

The ethanol extracts exhibited high contents of phenolics and high radical scavenging activity (DPPH scavenged, as a percentage). The results strongly suggest that phenolics and flavonoids are important components of the *H. erinaceus* extracts, and this could explain their high radical scavenging activity. Extracts of *H. erinaceus* are free radical inhibitors or scavengers, acting possibly as primary antioxidants. It has been reported that antioxidant effects of a compound may be concomitant with the development of reducing power. Reducing properties are generally associated with the presence of reductones, which have been shown to exert antioxidant action by breaking the free radical chain via the donation of a hydrogen atom [48]. Conversely, in the DPPH radical investigation, the results suggested that some other antioxidant compounds, not only total phenolics, are responsible for reductive capabilities. Overall, from this analysis we concluded that *H. erinaceus* can control oxidative damage and can act as an antioxidant. Ultrasonic extraction of *H. erinaceus* with ethanol resulted in relatively good antioxidant activity concerning the extracts described in these previous reports. Accordingly, Gursoy et al. [49] noted that the radical scavenging activity on DPPH of methanol extracts from *M. rotunda* at 2 mg/mL was 33.94 ± 0.96%. The variation of the antioxidant activity in many scientific articles can also be attributed to the solvent used [50].

Moreover, our results are in accordance with literature data for other *Hericium* species [44]. HPLC analysis showed erinacine A as the main compound in our ultrasonic-derived alcoholic extracts, suggesting that it may also be responsible for its antioxidant effects. The yield rate of erinacine A in the *H. erinaceus* after ethanol extraction was ~4 mg/kg, as confirmed and quantified by the HPLC procedure [18].

The chemical composition of mushrooms varies according to species [51,52]. Among the *Hericium* spp., *H. erinaceus* is the most studied. Its fruiting body is known as a good source of carbohydrates (76.5% DW, dry weight), protein (18.8% DW), ash (7.52% DW), fiber (7.10% DW), and fat (2.01% DW). These mushrooms also contain several amino acids, substantial amounts of potassium and phosphorus, and aroma substances [52]. The proximal composition of *Hericium erinaceus* can be seen in Table 3. *H. erinaceus* contains high energy, proteins, total antioxidant capacity (TAC), fiber, and fat. In the literature, arabinose has been evaluated as one of the minor sugars in mushrooms [36], but for the *H. erinaceus* studied herein, arabinose was the major sugar, and this can be attributed to the protocol followed for the development of the *H. erinaceus* mycelia. A large amount of arabinose is a characteristic of the genus *Hericium* [53,54]. The nutritional composition of *H. erinaceus* biomass can also be related to the composition of fat, protein, and carbohydrates used for cultivation in the glass vessel.

However, the inhibitory activity of each ethanol extract of *H. erinaceus* toward AChE was significantly (*p* < 0.001) lower than that of galantamine, the positive control. A previous study reported that the percentage of acetylcholinesterase inhibition activity in the fungal species *Pleurotus pulmonarius* ranged from 57.24 to 69.05% at concentrations of 0.063–1.0 mg/mL [48].

Diverse studies have reported that edible mushrooms possess bioactive compounds that enhance various antioxidant enzymes in the body [1]. Over the past decade, *H. erinaceus* has attracted increasing attention in the fields of functional foods and biomedicine. Numerous studies have reported the potential medicinal values or health-beneficial activities of this mushroom, like anti-cancer, anti-hypertensive, hypolipidemic, and neuronal disease protecting activities [1,11]. This study also established a good relationship between radical scavenging activity and phenolic/flavonoid compounds, thus suggesting a potential neuroprotective effect in AD.

## 5. Conclusions

This is the first study to investigate the antioxidant potential of *Hericium erinaceus* extracts obtained by ultrasonic extraction with ethanol. Based on our study, we can conclude that mushrooms seem to be a potential natural source of dietary flavonoids and polyphenols, displaying a great range of compounds in valuable concentrations. These results indicate the medicinal and antioxidant properties of *H. erinaceus* for use in alternative medicine. The methodology realized the development of *H. erinaceus* biomass in a controlled in vitro medium and its subsequent submission to the ultrasonic extraction technique, which can enrich the isolation of antioxidants in *Hericium erinaceus*, particularly polyphenols and flavonoids correlated with the diterpenoid erinacine A, known for its high antioxidant activity. The optimized extraction conditions were 80% ethanol, extraction time of 45 min, and solvent–material ratio of 1:30 (g/mL). The total content of phenolics in this optimized *H. erinaceus* extract was 23.2 mg GAE/g DM, and in the DPPH test, the antioxidant activity reached an IC_50_ of 87.2 µg/mL. According to the results above, *H. erinaceus* biomass may be introduced as a possible valuable source of nutritive supplements exhibiting antioxidant properties. Our future studies are ongoing and will focus on researching the possible beneficial effects on the brain of *H. erinaceus* extracts.

## Figures and Tables

**Table 1:** Box–Behnken Design (BBD) experimental design with the independent variables and observed responses.

Run Order ^a^	Ethanol% (X_1_)	Extraction Time (X_2_) (min)	Solvent-to-Material Ratio (X_3_) (mL/g)	Yield%	TPC ^b^ (mg GAE/g DM)	TFC ^b^ (mg QE/g DM)	DPPH Assay ^b^ IC_50_ (µg/mL)
1	40	20	20	9.30	11.1 ± 0.56	1.98 ± 0.01	55.3 ± 0.32
2	80	20	20	15.20	21.2 ± 0.65	3.11 ± 0.01	85.4 ± 0.15
3	40	45	20	10.50	13.3 ± 0.32	1.88 ± 0.01	56.3 ± 0.27
4	80	45	20	17.50	22.2 ± 0.29	3.25 ± 0.01	92.4 ± 0.37
5	40	30	10	9.60	12.8 ± 0.18	1.20 ± 0.01	49.3 ± 0.29
6	80	30	10	16.20	22.3 ± 0.33	2.65 ± 0.01	88.2 ± 0.21
7	40	30	30	9.20	11.1 ± 0.14	0.65 ± 0.01	51.6 ± 0.36
8	80	45	30	18.00	23.2 ± 0.11	3.26 ± 0.01	87.2 ± 0.77
9	60	20	10	13.30	13.5 ± 0.65	0.96 ± 0.01	65.1 ± 0.54
10	60	45	10	13.20	14.2 ± 0.54	0.85 ± 0.01	61.1 ± 0.11
11	60	20	30	13.10	14.1 ± 0.64	0.75 ± 0.01	63.8 ± 0.91
12	60	45	30	12.90	13.2 ± 0.26	0.84 ± 0.01	59.2 ± 0.46
13	60	30	20	12.90	13.6 ± 0.45	0.59 ± 0.01	52.1 ± 0.61
14	60	30	20	12.60	13.2 ± 0.19	0.59 ± 0.01	52.6 ± 0.45
15	60	30	20	12.60	12.6 ± 0.57	0.61 ± 0.01	49.8 ± 0.32
16	60	30	20	12.50	13.2 ± 0.75	0.61 ± 0.01	52.6 ± 0.41
17	60	30	20	12.50	13.6 ± 0.94	0.59 ± 0.01	52.8 ± 0.67

^a^ Run order—randomized. ^b^ The values are given as mean ± standard deviation of triplicate determinations; total phenolic content (TPC) expressed as mg GAE/g DM; total flavonoid content (TFC) expressed as mg QE/g DM; free radical scavenging activity expressed as µg/mL; DM: dry matter.

**Table 2:** Estimated regression model of the relationships among response variables (yield, TPC, TFC, and DPPH of *H. erinaceus* extracts) and independent variables (*X*_1_, *X*_2_, *X*_3_).

Variable	*p*-Value			
	Yield	TPC	TFC	DPPH
*β_0_*	< 0.001	< 0.001	> 0.001	< 0.001
*X_1_*	< 0.001	< 0.001	> 0.001	< 0.001
*X_2_*	0.0352	< 0.001	> 0.001	< 0.001
*X_3_*	< 0.001	< 0.001	> 0.001	< 0.001
*X_1_^2^*	0.0312	< 0.001	> 0.001	< 0.001
*X_2_^2^*	0.217	< 0.001	> 0.001	< 0.001
*X_3_^2^*	0.281	< 0.001	> 0.001	< 0.001
*X_1_X_2_*	3.36	< 0.001	> 0.001	< 0.001
*X_1_X_3_*	10.3	< 0.001	> 0.001	< 0.001
*X_2_X_3_*	12.5	< 0.001	> 0.001	< 0.001

**Table 3:** Proximate composition (g/100 g dw), free sugars, and fatty acids in *H. erinaceus*.

Mushroom	Crude Protein (%)	Crude Fat (%)	Fiber (%)	Carbohydrate (%)	Arabinose (%)	Mannitol (%)	Ash (%)	Energy Kcal/100 g (%)
*H. erinaceus*	15.04 ± 0.20	2.03 ± 0.40	6.12 ± 0.20	77.23 ± 0.30	16.46 ± 0.20	5.26 ± 0.20	6.53 ± 0.20	375.30 ± 0.10

Values are expressed as mean ± SD of carefully conducted triplicate experiments.

**Table 4:** Macro- and microelements in the *Hericium erinaceus* biomass developed in the bioreactor.

Macroelements (mg/100 g dw)	*Hericium erinaceus*
**Ca**	42.32 ± 2.36
**Mg**	74.54 ± 3.14
**Na**	546.23 ± 3.10
**K**	1030.03 ± 12.10
**Microelements (mg/100 g dw)**	
**Fe**	5.23 ± 0.02
**Cu**	0.14 ± 0.02
**Mn**	0.04 ± 0.01
**Zn**	2.13 ± 0.03

Values are expressed as mean ± standard deviation of three replicate determinations.

**Table 5:** Reducing power of *Hericium erinaceus* extract.

	Absorbance Values (at 700 nm) at Different Concentrations (mg/mL)			
	0.05	0.10	0.50	1.00
*H. erinaceus* extract	0.020 ± 0.004	0.024 ± 0.021	0.021 ± 0.006	0.065 ± 0.015
Quercetin ^a^			2.501 ± 0.052	
Butylated hydroxyanisole (BHA) ^a^			2.401 ± 0.016	
Ascorbic acid ^a^			2.126 ± 0.127	

^a^ Positive controls.

**Table 6:** Acetylcholinesterase and butyrylcholinesterase inhibitory activity levels of the 80% ethanolic *H. erinaceus* extracts.

Run Order	Extraction Time min	AChE Inhibition % (1 mg/mL) ^a^	BChE Inhibition % (1 mg/mL) ^a^
*1*	20	51 ± 1.5	48 ± 1.4
*2*	30	52 ± 1.4	48 ± 2.5
*3*	45	53 ± 2.5	49 ± 3.1
			
Galantamine	-	73 ± 2.6	62 ± 3.2

^a^ Inhibition percentages represent the mean ± standard deviation of three parallel measurements (*p* < 0.05).

## Supplementary Materials

The following are available online at https://www.mdpi.com/2304-8158/9/12/1889/s1, Figure S1: Erinacine A calibration curve for the HPLC method.
