# Supplementary material for: Optimization of Ultrasonic Extraction to Obtain Erinacine A and Polyphenols with Antioxidant Activity from the Fungal Biomass of Hericium erinaceus
Source: Foods. 2020 Dec 18;9(12):1889. doi: 10.3390/foods9121889 (PMC7766035; doi:10.3390/foods9121889)
Supplement: Supplementary file 1 [file foods-09-01889-s001.pdf]

foods

Supplementary materials

Optimization of ultrasonic extraction to obtain Erinacine A and polyphenols with antioxidant activity from the fungal biomass of *Hericium erinaceus*

Mihai-Vlad Valu [1,2], Liliana Cristina Soare [1], Nicoleta Anca Sutan[1], Catalin Ducu [2], Sorin Moga [2], Lucian Hritcu [3], Razvan Stefan Boiangiu[3] and Simone Carradori [4,*]

[1] University of Pitesti, Faculty of Science, Department of Natural Sciences, Targu din Vale Str., 110040 Pitesti, Romania; mihai.valu@upit.ro (M.V.V.); cristina.soare@upit.ro (L.C.S.); anca.sutan@upit.ro (N.A.S.)

[2] University of Pitesti, Regional Research and Development Center for Innovative Materials, Products and Processes from Automotive Industry, 11 Doaga Str., 110440 Pitesti, Arges, Romania; catalin.ducu@upit.ro (C.D.); sorin.moga@upit.ro (S.M.)

[3] Alexandru Ioan Cuza University of Iasi, Department of Biology, Bd. Carol I, No. 11, Iasi 700506 Romania; hritcu@uaic.ro (L.H.); razvan.boiangiu@student.uaic.ro (R.S.B.)

[4] G. d'Annunzio" University of Chieti-Pescara, Department of Pharmacy, Via dei Vestini 31, Chieti 66100, Italy; simone.carradori@unich.it (S.C.)

* Correspondence: simone.carradori@unich.it

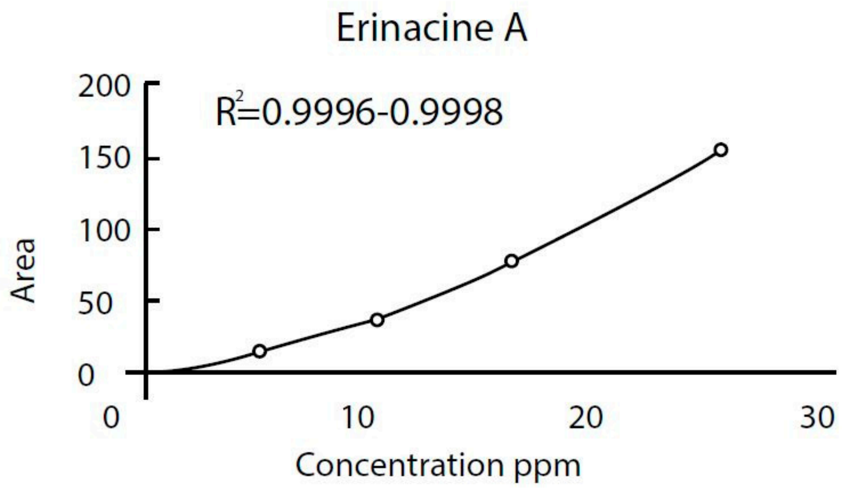

Figure 1. Erinacine A calibration curve for the applied HPLC method.
